# Ten future challenges for synthetic biology

**DOI:** 10.1049/enb2.12011

**Published:** 2021-08-02

**Authors:** Olivia Gallup, Hia Ming, Tom Ellis

**Affiliations:** ^1^ Department of Bioengineering Imperial College London London UK; ^2^ Imperial College Centre for Synthetic Biology Imperial College London London UK

**Keywords:** automation, biosensors, bio‐economy, genome engineering, industry, microbial engineering, synthetic biology, artificial life, biotechnology, genomics, learning (artificial intelligence), reviews, sustainable development

## Abstract

After 2 decades of growth and success, synthetic biology has now become a mature field that is driving significant innovation in the bioeconomy and pushing the boundaries of the biomedical sciences and biotechnology. So what comes next? In this article, 10 technological advances are discussed that are expected and hoped to come from the next generation of research and investment in synthetic biology; from ambitious projects to make synthetic life, cell simulators and custom genomes, through to new methods of engineering biology that use automation, deep learning and control of evolution. The non‐exhaustive list is meant to inspire those joining the field and looks forward to how synthetic biology may evolve over the coming decades.

## 1 INTRODUCTION

An old British saying is that when you turn 21, you get the keys to the door. This year synthetic biology turned 21, and it certainly feels like the subject now owns a set of keys for unlocking bioscience and the bioeconomy. Multiple synthetic biology companies have debuted on the stock market with billion dollar valuations, genome engineering has permeated into every space of bioscience research, and writing, editing and recoding DNA is now helping to solve long‐standing genetic diseases and is a key part of the rapid rollout of vaccines fighting the Covid pandemic [1, 2]. With the subject now a demonstrated success, where can it go next and what bigger challenges can it take on? A decade ago the field was challenged with a list of hard truths as to what would be needed next [3], but most of these have now been overcome and what is possible to build is rapidly expanding [4]. So here, we instead now look forward to 10 major research advances that we personally expect and hope to see come from synthetic biology. Many of these advances are already well underway, thanks to vibrant communities who are beginning to make progress towards grand challenges in these themes.

## 2 AUTOMATION AND INDUSTRIALISATION

From the outset, synthetic biology has pushed for standardisation of biological parts for two reasons: reusability and ease of DNA assembly. Such standardisation has the downstream benefit of enabling automation of DNA construction and scale up of this operation to make large numbers of engineered cells that can be tested in parallel. This ‘industrialisation’ of the process of building and testing is something the field has long pursued but still is not commonplace in most research groups. So how will this change in the future?

Tools for automating the Design–Build–Test–Learn (DBTL) cycle are already mostly in place, especially in biofoundries and at major companies [5]. Repositories of characterised parts in addition to various computer‐aided design (CAD) tools facilitate selection of parts and design of genetic constructs. Liquid‐handling robots capable of transferring micro‐, nano‐ and even picolitres of reagents with high accuracy and rapid throughput streamline complex combinatorial experimental setups and improve experimental reproducibility. Data handling tools codify experimental setups and collate experimental metadata, ensuring traceability of errors that facilitate debugging. Statistical analysis software parses large quantities of data to generate insight and Design of Experiments (DoE) strategies help to optimise future experiments, thereby closing the DBTL loop [6].

The next decade will see increasing efforts to piece together these tools into efficient and versatile pipelines to generate synthetic biology solutions at breakneck speed. Concomitant is the growing digitalisation in the new age of synthetic biology and the need for better data infrastructure, management and curation. Standardisation of data exchange formats, ontologies, protocol description, experimental data and metadata reporting will not only facilitate the flow of information along the DBTL pipeline and transferability of data between different platforms but also enable better open‐source software development and data sharing between different research groups and within companies. Alongside this, automation will also push (and pull) the need for standardisation of genetic parts and measurement of their performance; both long‐term goals of the field that enable predictability. It will also likely lead to better standardisation of the host cells and strains used as the main chassis organisms at different biofoundries.

The fastest progress on this challenge is now happening at a dozen or so biofoundries and well‐funded companies who have the necessary spending, development and integration required to make automation work. Biofoundries and companies operating as ‘cloud labs’ promise to offer their software and platforms to partners and researchers to help accelerate the iterative DBTL cycle and shorten the development time for the whole field [7]. Cloud labs allow those without synthetic biology expertise and appropriate lab facilities to quickly kick‐start projects of interest in the field, have strains improved by engineering or run large‐scale screening experiments. Ginkgo Bioworks and Microsoft's Station B are test cases for the integrated biofoundry model of synthetic biology research and development, while companies such as Synthace and Riffyn occupy niches in laboratory automation and data management. The aim in all the cases is to focus project work into learning and design, rather than building and testing, which is something the field has long desired. The problem for synthetic biology, however, is whether this advance can be done democratically. It may be more financially beneficial for companies in this space to compete as expert engineering contractors and so close access to their platforms and knowledge bases rather than open them up to all. Academic biofoundries also face the challenge that centralised experiments and lab work via the cloud do not align with the traditional institutional view of PhD students and postdocs working all day at the bench on their project. Such an approach therefore requires a rethinking of the classic scientific project. Indeed, those helping to run and fund synthetic biology in academia and in industry will need to take brave decisions to help switch the field to this more efficient way of working in the future.

## 3 DEEP LEARNING FOR DNA DESIGN

Machine Learning, Artificial Intelligence (AI) and Deep Learning are unavoidable terms these days in journal papers, grant proposals and start‐up pitches, and so you will not be surprised to learn that everyone is trying to add this element to their research, with mixed success. Notable early wins in bioscience have used deep learning to classify microscopy images to help predict protein structure and to predict drug molecular structures that work as antibiotics [8, 9]. But where deep learning is likely to have the biggest impact for synthetic biology is in DNA design. Why? Because writing genetic programmes in DNA is a language problem.

Designing DNA is at the crux of much of synthetic biology. Yet, editing even a simple set of genes can quickly lead to a headache for bench scientists. The beauty of deep learning is the ability to transform one type of data into another without knowing the exact details of the conversion. Even physical constraints can be learnt with the right sort of data, allowing some slack if some fundamental knowledge of the system is missing. The highly acclaimed natural language processing (NLP) model GPT‐3 [10] has sparked serious ethical debate due to its ability to generate highly convincing ‘human’ text, even when given only a few learning points as input in a process called few‐shot learning. This showcases the power that deep learning networks can lend to the more complex language task of interpreting and generating DNA sequences.

Compared to previous disciplines, synthetic biology brings its own advantages to the DNA design table. Instead of solely reading in collected data, reading *and* writing are both possible, thanks to advances in genome editing and DNA synthesis. This means that more meaningful training data can be generated to pressure‐test a model's internal representation of a system and embed a deeper understanding. Active learning is a machine learning paradigm that helps determine the best next set of perturbations to supplement a learning model and can easily be integrated into automated workflows. Several biotech companies have already deployed such methods to trawl biological sequence space in a targetted manner. Built‐in data curation also reduces the volume of training data that a model needs to achieve the same performance.

In the future, therefore, deep learning models could help us move away from treating DNA sequences as precious nature‐crafted parts, which we standardise and used as modules in biofoundry‐made assemblies. Instead, deep learning should allow us to write optimal DNA sequences for certain combinations of genetic parts and genetic contexts simply based on high‐level commands. Deep learning itself may also benefit from what we understand about natural evolution of DNA sequences towards more optimal performance. The trial‐and‐error process of mutation towards improvement is captured in machine learning, but what about other features of biological evolution such as error detection and correction and sequence recombination? As we better understand how to guide DNA evolution (as discussed below), we can also expect benefits from this to feedback to give better design.

## 4 DESIGNING WITH WHOLE‐CELL SIMULATIONS

The amount of data associated with biology is increasing exponentially each year, as ‘omics’ methods gather thousands and millions of data points on cells, genes, transcripts and proteins with each experiment. Nearly a decade ago, researchers focussed on natural minimal cells took the first steps in exploiting such data to make a simulation of how all the genes and proteins behave in *Mycoplasma genitalium*, a bacteria with a genome of only around 500 genes [11]. They developed mathematical models of all key processes in the cell, parameterised these using omics data, and then devised a method to integrate these models into a dynamic simulation of a cell cycle. This feat of systems biology helped bring new understanding to the resource use of cells, and perhaps most excitingly for synthetic biology it was able to predict how the organism was affected when genes were deleted from the genome or introduced into this cell [12].

Now imagine what would be possible if the gigabytes of omics data available online could be used to make similar or better simulations of cells and their functions for commonly engineered model organisms. In theory this could change the whole process of design in synthetic biology, allowing projects with gene circuits and metabolic pathways to be designed, modelled and optimised first for function (as is currently done in many projects), before being simulated for their expected performance and impact within the environment of the host cell. The ‘host cell dimension’ would become a tractable parameter for model‐based design, allowing synthetic biologists to better consider knock‐on effects of engineering within a cell, such as resource use, metabolite fluxes, and retroactivity in gene regulation [13]. To some extent, progress in systems and synthetic biology is already close to this aim. Genome‐scale metabolic modelling is now routinely used to advance biosynthesis cell engineering projects in many microbial systems, and the most advanced of these models include within them the resource cost of expressing the enzymes [14]. Whole cell simulations are the logical extension of these models and would allow the approach to be used beyond metabolic engineering projects into more complex synthetic biology projects, such as applications of logic circuits where changes in transcription factor levels are important.

If the approach used in *Mycoplasma* proves scalable, we can look forward to a future where whole‐cell simulations exist as a design tool for organisms like Baker's yeast and human cell lines. However, in the immediate future a whole‐cell simulation of *Escherichia coli* is the most anticipated. It would provide the first real test for synthetic biologists on how to design engineered strains and genetic constructs with such a simulation in place and ideally reduce the experimental trial‐and‐error of engineering cells.

However, we also need to realise that simulations do not always give correct predictions for what happens in an actual experiment. Simulations would likely start only as a way to narrow down design space, but hopefully as more people use and test their capabilities they would improve in predictive power. Simulations are also likely to be critical for efforts to design and build custom genomes and cells from scratch. Access will be a major issue as well. Running a cell cycle of *M. genitalium*'*s* ∼500 genes is already computationally demanding, and as gene numbers go up (1000+ for *E. coli*) there is the prospect of exponential network complexity leading to an explosion in the computation time to run a single simulation. How such complex simulations can become an accessible design tool for the community will need to be carefully considered as this advance progresses.

## 5 BIOSENSING: DETECTING ANYTHING, ANYWHERE

Nature is replete with information that living things naturally sense and respond to and co‐opting these mechanisms into engineered cells to make biosensors has been a staple of synthetic biology projects for 20 years now. But if you consider the ubiquitous, diverse and incessant sensing that biology is performing all over our planet right now, then it feels like biosensing can do so much more. Sensing by living cells is a superpower that humankind should be doing everything it can to understand and exploit. Being able to detect anything, anywhere would revolutionise our world in so many ways; not just in research but in pandemic preparedness and assessing our own health and the health of our planet as well.

How can we get to a generalisable and modular architecture for biosensing that will allow us to sense anything? Already we make biosensors using proteins that bind molecules of all shapes and sizes, and more recently, we have made biosensors that detect DNA and RNA sequences via toehold switches and CRISPR enzymes [15, 16]. Improving the characterisation of natural proteins, oligonucleotide ligands and other macromolecules is a great start; it will vastly expand the repertoire of targetable analytes for detection. Several landmark studies have recently shown that generalisable protein‐based sensors are a possibility [17, 18, 19] and riboswitch design and directed evolution are beginning to become established as well. Furthermore, significant strides are being made in protein structure prediction by AlphaFold, Rosetta, RaptorX and others [9, 17, 20, 21]; so in the future, we should be able to design and tune the sensitivity and specificity of proteins and RNA to sense a myriad of ligands.

Differential gene expression of organisms in response to diverse stimuli can also be used as a means of indirectly capturing complex information about the environment a cell encounters without the need to make a single specific sensor for a ligand. Analysing the transcriptomic state of cells to learn signature expression profiles of different ‘encounters’ can give us a route to identify the sensing of things for which no single biosensor exists. In many ways, this would be similar to what we do when we smell food, with sensors for many different molecules in our nose leading to our brains detecting a signature pattern for a more complex stimulus. Genetic logic circuits and recombinase‐based memory systems could be engineered into cells to respond to transcriptome signature patterns to convert these transient encounters into recorded detection and read out.

The more challenging question is how to widely deploy and easily read out from biosensors. The first consideration is the longevity of the biosensing system. Efforts to encapsulate or lyophilise whole‐cell or cell‐free biosensors have prolonged the lifespans of biosensors and allowed transport without a cold chain [22]. Engineering sensing motifs into naturally stress resistant biological constructs such as spores and biofilms can also enable sensing under harsh environmental conditions [23]. The second consideration is the ease of use of the biosensor. An ideal biosensor would require minimal pre‐ and post‐processing for sensing and reporting; it should be autonomous and easy to deploy. The third consideration is the acquisition of data from biosensors. Colorimetric reporting is probably the most useful for point‐of‐care diagnostics. Smartphones with powerful image capturing and processing abilities are now commonplace, and these could facilitate the on‐site analysis of multiplexed biosensing arrays with colorimetric output. Relaying information from the biosensor to external electronic units is perhaps the long‐term goal, as this will facilitate real‐time monitoring of biology, linking this directly to our electronic world. Physical bio‐to‐electro interfaces do not necessarily need to exist to achieve this. For example, plants and soil in fields could emit signals that can be remotely recorded by multispectral imaging drones and satellites [24, 25].

## 6 REAL‐TIME PRECISE CONTROL OF EVOLUTION

The analogies and language of synthetic biology frame the research as the effort of engineering biology, like we would engineer a car or a computer. But nature is not an engineer; it is a tinkerer, using evolution to modify and improve biological systems. While conceptually simple, evolution is nature's ultimate technology achieving incredibly complex systems that are optimised for their environment without the need for any careful design and planning. It is a defining feature of biology that we should aim to incorporate more into the way we engineer cells and use them in applications in synthetic biology.

Currently, most synthetic biology projects work to design and construct a cell or strain and then put it to work, performing a task like biosynthesis or biosensing. We either just hope that mutation and selection will not act upon our ‘finished product’ once it is in operation, or in some cases we design it as best as we can to reduce this chance [26]. But living matter is inherently dynamic, with mutation and selection ever‐present. As such evolution—even on small scales in well‐controlled environments—is something that cannot be ignored and indeed cannot be completely prevented. Instead, we need to learn to harness evolution when engineering biology, working out how to control and guide the fate of engineered genes, cells and organisms in different environments and for different applications.

The biotechnological tools needed for this advance are rapidly emerging. CRISPR‐based systems and related advances like MAGE allow us to design, direct and control mutation to certain sites and genes within living systems [27, 28, 29]. Deep‐learning models are also enabling prediction of how DNA and amino acid changes affect genes, gene regulation and protein shape and function. Directed evolution has already proved its value in producing hundreds of improved and novel enzymes for adapting hosts for better metabolic biosynthesis and even for optimising gene circuits [30]. And now molecular tools for continual in vivo directed evolution of target genes also exist [31, 32, 33, 34, 35]. It is not a great leap to imagine engineered cells built to have externally controllable evolution of target genes and chromosomes so that we can optimise them as and when, either to adapt them to new tasks and environments or continually improve them from an initial prototype design. Who would not want to have a system where synthetic genetic constructs could be built quickly as prototypes and then would evolve to self‐optimise once put to use?

Engineering biology *with* evolution, rather than despite it, is clearly a very attractive prospect, but it will require a radical new way to think and design [36]. Synthetic biologists are always keen on characterising, predicting and standardising for measurable outcomes of DNA design like target protein levels, metabolite yields and exponential growth rates; so how will we design, predict and measure for ‘evolutionary potential’? A paradigm shift in thinking will be needed to move to a design approach that takes into account a whole new dimension, but if we can make progress in this, the entire way that synthetic biology works 20 years from now will be revolutionised.

## 7 CELLULAR COMMUNITIES AND MULTICELLULARITY

A grand challenge for synthetic biology is to design the differentiation and specialisation of cells to enable effective and fruitful ‘division of labour’ in synthetic multicellular systems. So far, synthetic biology has focussed largely on each cell in a population being engineered with a single task. But following initial projects on themes like programmed edge detection in biofilms and population‐based oscillators, teams have since reported work towards more complex multicellular functions, including consortia‐based computation, molecular Turing patterns, and synthetic morphogenesis [37].

Quorum sensing systems have so far been the method of choice for communication between cells that allow for temporal, dynamic and orthogonal levels of control. Indeed, the AHL‐mediated quorum sensing system in bacteria has been exploited in many studies. In eukaryotes, engineered cell‐to‐cell signalling can be transduced by auxins or by GPCR‐based signalling, and in mammalian cells engineered modular sensors based on the Notch signalling system have recently proven to be particularly powerful [38]. There are few barriers in determining what can constitute a communication channel, and even DNA itself has been used for this task [39]. But what is most useful for the field right now are modular toolkits of genetic parts that enable reliable, dynamic and orthogonal cell‐to‐cell communication among many cells. However, these alone will not enable predictable and robust engineering of consortia and multicellular systems. New considerations like growth competition, symbiosis, cell cooperation, dormancy and escape mutation will become just as important as we try to engineer cells doing different tasks to grow and work together correctly.

Similar to how different neural networks interface and support each other in composite learning, co‐cultures of engineered cells offer opportunities. For example, they can make the production of natural compounds more efficient by having different strains act as functional modules that individually do not shoulder the whole burden of a costly function, such as complex metabolic pathways like that of rosmarinic acid [40]. A ‘cells as modules’ approach could offer plug‐and‐play organisms and consortia that can be thrown into new systems, like reusable functions in computer code. Realising such systems will require work to define the compatibility of dynamic ranges in communication elements and also their universality and composability.

Engineering systems with multiple interacting cells clearly offer huge power for advancing synthetic biology, but these do not have to be limited to just co‐cultures or co‐dependent communities. The complexity, robustness and multifunctionality of natural multicellular organisms like plants and animals already show us the opportunities offered in systems where cells physically attach together and differentiate to do specialist tasks in tissues, organs and bodies. Our ability to write synthetic differentiation programmes from DNA parts is progressing [41], and we are beginning to be able to direct cells to attach together and grow in distinct patterns as well [42, 43]. As we progress towards predictable engineering of co‐cultures and consortia, it would be ideal to also develop the rational engineering of proto‐tissues and organoids in parallel so that advances in one can crossfeed into the other.

## 8 CUSTOM AND DYNAMIC SYNTHETIC GENOMES

Perhaps the most obvious next area for synthetic biology to tackle at scale is synthetic genomes. The first cell living with a synthesised genome was achieved over 10 years ago by the J Craig Venter Institute. Since then a minimised *Mycoplasma* genome of only essential genes has been produced, multiple engineered yeast chromosomes have been completed and most recently a synthetic version of the *E. coli* genome that only uses 61 of the 64 codons has been made [44, 45, 46]. A standard approach to making synthetic genomes has started to emerge and the DNA costs and work required, while still tremendous, have fallen enough for megabase chromosomes to now be made by individual teams [47]. It will not be long before bacterial and yeast chromosome synthesis projects become the goal of individual groups, and within the next decade synthetic genomics will also be starting to tackle the much larger genomes of multicellular organisms like mammalian cells and plants [48].

Most current synthetic genome projects aim to deliver new knowledge, giving us new insights into genome coding, content and organisation; aspects that are difficult to unpick by other methods. However, in the future, with cheaper DNA synthesis and automated and scalable DNA assembly, making synthetic genomes designed for applications will become the new challenge [49]. Already genomes with reduced codon usage are customised for applications of genetic code expansion; particularly adding‐in non‐canonical amino acids to the proteins made by the cell [50]. Genomes designed to be specialised for other applied reasons, such as one customised for streamlined metabolism for biosynthesis projects, are likely to follow next. As the field tackles larger genomes of multicellular organisms, it may be desirable to customise these to focus only on the genes required for specific roles. Cell‐based therapies are increasingly popular, for example, but for safety reasons it could become more desirable in the future to make these from cells with minimal synthetic genomes so that genes not required for therapy function are removed and do not pose any unintended risks.

Designing custom genomes, however, is not going to be a straightforward task. Whole‐cell simulations and deep learning from omics experiments will hopefully soon bring us closer to a time where we are better equipped to de novo ‘compose’ the set of required genes and regulatory sequences required to write a novel custom genome that makes a functional cell. Perhaps ahead of that time, will come a more radical idea, dynamic genomes, where we intentionally include recombinases and their binding sites along with other mutation and DNA fusion hotspots into existing genomes and then trigger these genomes to physically change over time to perform in new ways. Genomes could be engineered to streamline themselves for specific fixed tasks as their cells differentiate, for example, by deleting regions and genes no longer required for their roles. Directed evolution and dynamic synthetic genomes are two powerful approaches that may well go hand‐in‐hand in the near future, and an inducible recombination system built into synthetic yeast chromosomes is already demonstrating the power of this approach [51].

## 9 ARTIFICIAL CELLS

While considerable effort has gone into synthesising new genomes for existing cells, a more fundamental approach to synthetic cells has been emerging from diverse corners of chemistry and biochemistry research that seeks to recreate the basic functions of a living cell purely by combining biochemical components. The goal is to produce artificial cells from the bottom‐up to achieve programmable synthetic mimics of the basic cells we see in biology. This may seem like a tall order, but the ground covered in artificial cell research over the last decade has solved a host of practical issues and has now opened up a fertile space for increasingly complex and interesting projects [52, 53, 54, 55, 56].

Understanding by constructing has been a motivating philosophy throughout synthetic biology and is the main driver in artificial cell research. Achieving a synthetic cell will help us define the barrier between the non‐living and the living as we create a self‐replicating entity from inert molecules. But on a more pragmatic level, it will also help us better understand how natural cells work. Using artificial cells, like lipid vesicle protocells, we can already study hard‐to‐assess aspects of molecular biology, such as the minute effects of typically confounding elements in a cell, like molecular concentration variations and macromolecular crowding. With the acceleration of advances in supporting fields like microfluidics, chemical compartmentalisation, and DNA synthesis, it is an exciting time to perform synthetic biology in protocells, and this field promises to mature and help us better interrogate the engineerability of simplified biological systems.

While achieving large‐scale biological behaviours (like a cell cycle) remains tricky in protocells, researchers are getting creative with the microstructures built to achieve functions like phagocytosis, cell‐to‐cell communication and even self‐replication, the latter representing one of the greatest challenges [57]. Giant lipid vesicles have paved the way for experiments with the construction of early cell‐like capsules and encapsulation of functional agents. Vesicles as artificial organelles have also inspired the blending of synthetic and natural components in cells, such as liposomes functioning as semi‐artificial bioreactors. Such modules are further useful for developing new experimental methods like cell communication with novel chemical messengers [58]. In general, compartmentalisation fosters regions in biological space free of confounding noise that can be experimented with to create systems that can compute, self‐assemble, and one day be able to achieve autonomous self‐replication.

As the diverse achievements in artificial cell research begin to mount up, it is only a matter of time before teams begin to collaborate to construct a cell and understandably several national and international consortia have now formed to tackle this grand challenge, including Build‐a‐Cell (United States) https://www.buildacell.org/ and SynCellEU (Europe) https://www.syntheticcell.eu/. It is therefore wise to start considering what artificial cells can be used for once they have become a reality. For some applications in synthetic biology, it is conceivable that protocells with customisable living features may eventually overtake natural microbes as engineering systems of choice. Beyond being a vehicle for us to understand the fundamental mechanisms of life, synthetic cells could soon be deployed as their own programmable systems, potentially being a tool that can take the best technologies of biology—evolution, autonomy, and self‐regeneration—and mix that with technologies and functions we have and use from non‐living systems in our everyday lives.

Artificial cell research also ties in closely with the recent push to develop biology that does not exactly follow the same rules as the central dogma: so‐called ‘xenobiology’ [59, 60]. Efforts to modify cells to use non‐standard chemical building blocks in DNA, RNA and peptides have proved successful at small scale in the last decade and are sure to be bearing more fruit soon. It will be particularly exciting to see non‐standard nucleic acids, flipped sugar and peptide chirality and different codon schemes being realised, and artificial cells will probably provide the ideal vehicle for first achieving this.

## 10 MATERIALS WITH DNA‐ENCODED PROPERTIES

A natural follow on from cellular communities is the burgeoning field of engineered living materials (ELMs) [61]. Appropriating cells as chemical factories has driven some of the biggest successes in the synthetic biology industry and fostered development at intersections with other fields, such as the food and fashion industries. Materials such as bacterial cellulose, spider silk and mushrooms are actively being used as the base for sustainable textiles, furniture, and even building materials in advanced architecture. Moreover, the range of materials that can be produced by microbes at scale is advancing, in no small part, thanks to imaginative iGEM projects. Besides *E. coli*, the workhorse organisms of synthetic biology and related bacteria such as *Gluconacetobacter* and *Bacillus* species are excellent producers of secreted cellulose and highly durable amyloid fibres [62, 63]. The expansion of genetic toolkits available for engineering material‐producing microbes will speed up the development times for diverse projects in the near future.

While a greener world composed of biologically produced material substitutes is within reach already, the true revolution underway is biology as a manufacturing discipline. Cells can not only be treated as miniaturised material factories (deployable virtually anywhere with the right conditions and media) but also be made to work together to modify a material on a molecular level, at almost no extra cost in terms of manufacturing techniques. Micropatterning, such as through optogenetics or cell‐to‐cell signalling, is a complex task for machines yet easy for millions of tiny microbes [64]. Such patterning can be very useful for healthcare applications or in advanced electronics where micrometre scale features are required. Similarly, chemical modifications that bestow advanced properties such as colour and hydrophobicity can be introduced as easily as inserting a bit of extra DNA at the right point within cells to trigger expression of functional proteins and enzymes. Of course, the manufacturing techniques that enable scalable production of newly developed materials will bring their own challenges, but once honed they will provide a competitive edge over prevailing materials with similar functions. This is already evidenced by the collaborations that several synthetic biology material startups have fostered with industry‐leading fashion brands that are trying to fix their carbon footprint.

These initial embellishments set the foundations for a future layer of complexity in ELMs, whereby engineered cells take on a second role within a material after it has been produced. We have seen such approaches in fields like soft robotics, where muscle‐like soft robots that can swim have been made using a gel‐like material containing engineered light‐responsive heart cells that beat at the trigger of a light pulse [65]. Such projects represent ELMs that are the most faithful to their name and promise to have a significant impact on the way we think about materials, what features we can introduce into them, and how we use them. Ideally, if synthetic biology can approach this major advance with a standardised framework, we can arrive at a point where the properties and functions of materials made in this manner can be defined and designed by modular DNA‐encoded programmes placed within the cells that make them. After all, we already see this in plants where the genome inside each cell contains the DNA programmes to grow and differentiate that cell and its surrounding tissue into diverse different materials from soft flower petals to hard nut shells.

## 11 ENGINEERED ORGANISMS FOR SUSTAINABILITY GOALS

Why pursue all these new advances, unless they can help us live better lives? Biotechnology has always had the power to improve our world, but in the last few decades, it has clearly focussed most of its efforts on helping humanity prosper and enabling us to live longer lives. An initial focus of using synthetic biology to improve our health is understandable; after all the first thing most people think of when we say ‘biology’ is our own bodies. But biology is much more than just human health, and working *with biology* over the next century will be the key to preventing climate catastrophe and ensuring that we can all live together on a healthy planet. Synthetic biology simply needs to be a major part of this solution.

Thankfully, sustainability is a trend that is already emerging and becoming established as a key goal for synthetic biology, especially in the newer generation of researchers. The global push towards building circular economies is clearly going to hinge on the biological transformation of industries, and so it is not surprising that people are looking to develop and employ engineered organisms that can help investors, companies and countries achieve their sustainability goals. Engineered organisms have the potential to create renewable sources of energy, enhance product life cycles, protect the environment and its biodiversity, increase food sustainability and improve health. Advances in synthetic biology are accelerating biological innovations to create a new value for current economies founded on environmentally sound principles. We are already seeing synthetic biology companies becoming established players in important areas such as nitrogen fixation, carbon and methane capture, plastics recycling, plant‐based products and pollution remediation. Plant‐based meat and lab‐grown meat are also major ways by which we can reduce our impact on Earth. These new, more sustainable and ethical ways of making food will require innovation in engineering biology to make them scalable.

The path forward, however, is plagued with many challenges. Is the public ready to accept engineered organisms as a ubiquitous part of modern life that helps our planet, rather than see them as further pollution of nature? Policy makers will need to have the prescience to garner support for bio‐based strategies from all levels of society. Many technical challenges also remain. In the energy and manufacturing sector, scaling up of biotechnological processes is still no easy feat. Expertise still needs to be built for efficiently transferring biological innovations from the laboratory scale to the industrial scale. Engineering organisms to subsist on cheaper feedstocks will also be required to bring down the price of biotechnology and make it the tool that can feed the world and recycle its materials. These are big challenges that will require nations and investors to take a long‐term position on, and researchers will have to work hard to match those ambitions over many years. The rewards, however, will be great—not just environmentally but likely also financially; so synthetic biologists everywhere should definitely get ready to rise to this challenge.

## 12 CONCLUSIONS

With these 10 advances in synthetic biology (and likely many others that will emerge), we hope that we can completely transform the way we work and live with biology on this planet in the near future. However, as has been mentioned already in some of these sections, maximising the impact of these scientific advances will be difficult without also tackling the social and political aspects that come hand‐in‐hand with new technologies and how they are used. Environmental bioengineering, designing organisms to evolve more rapidly and synthesising artificial living cells are all ideas that excite scientists but are likely to raise major concerns with a large section of the public. Perhaps the greatest future challenge will therefore be for everyone in synthetic biology to do their part in engaging with the public, listening to alternative viewpoints, adapting their ideas and making their work and intentions as open and available as possible from the start. When it comes to challenges as big as the planet's own well‐being, everyone on Earth is a stakeholder, and so all views are valid and should be considered. This is the only way that we can build engineered biology that will be accepted by the world and given the appropriate regulation that it requires for safe use. It is up to us and the generations below us to correctly develop these technologies and use them wisely, fairly and safely to protect and collaborate with nature, rather than exploit and deplete it. Synthetic biology may offer great promise for space exploration, but it will always have even more promise for helping us preserve the planet that we have all evolved for.

## CONFLICT OF INTEREST

The authors declare no conflicts of interest.

## Data Availability

Data sharing not applicable to this article as no datasets were generated or analysed during the current study.
